# Disrupting Othering: A Continuum of Age-Integrated Strategies for Gerontology Education

**DOI:** 10.1093/geroni/igab046.1361

**Published:** 2021-12-17

**Authors:** Lisa Borrero, Lisa Borrero

## Abstract

Given the extensive negative impacts of ageism, it is incumbent upon institutions of higher education to lead the charge in promoting age inclusivity and dismantling ageism, particularly in the classroom. Traditional teaching approaches are insufficient to meet the needs of our increasingly diverse student population or our aging post-COVID society. Gerontology classrooms require new, innovative, and purposeful ways of engaging students in course content. This symposium brings together faculty who intentionally promote age inclusivity by immersing students in age-integrated experiences to varying degrees and in different ways. These approaches fundamentally reject the idea of older adults as “other” by promoting an age-inclusive reality in which individuals of all ages are equally valued. Dr. Lyn Holley will describe a new course which requires projection of a future, older self and two systematic life interviews – one of a real US elder and one of an imagined counterpart in a different country; comparative analysis reveals how aging is shaped by society. Dr. Skye Leedahl will discuss challenging ageism by examining existing age-focused policies and programs and having students engage in six intergenerational discussions and reflective writing. Dr. Tina Newsham will describe a newly revised practicum in which immersive, real-world experiences with older adults are incorporated, along with meaningful reflection and the development of an e-portfolio. Finally, Drs. Laura Donorfio and Lisa Borrero will explain the ways “othering” is disrupted when students imagine themselves as older adults, and use creative approaches that demonstrate how to negotiate key aspects of the aging process.

